# Characterization of the gut microbiota in diabetes mellitus II patients with adequate and inadequate metabolic control

**DOI:** 10.1186/s13104-021-05655-z

**Published:** 2021-06-24

**Authors:** Angie Joyce Hamasaki-Matos, Katherine Marlene Cóndor-Marín, Ronald Aquino-Ortega, Hugo Carrillo-Ng, Cesar Ugarte-Gil, Wilmer Silva-Caso, Miguel Angel Aguilar-Luis, Juana del Valle-Mendoza

**Affiliations:** 1 School of Nutrition, Faculty of Health Sciences, Universidad Peruana de Ciencias Aplicadas, Lima, Peru; 2 Research and Innovation Centre of the Faculty of Health Sciences, Universidad Peruana de Ciencias Aplicadas, Lima, Peru; 3 School of Medicine, Faculty of Health Sciences, Universidad Peruana de Ciencias Aplicadas, Lima, Peru; 4 Laboratorio de Biología Molecular, Instituto de Investigación Nutricional, Lima, Peru

**Keywords:** Gut microbiota, Type 2 diabetes, A1C hemoglobin, PCR

## Abstract

**Objective:**

The objective of this study was to characterize the composition of the gut microbiota in type 2 Diabetes Mellitus (T2DM) patients with adequate and inadequate metabolic control, and its relationship with fiber consumption.

**Results:**

A total of 26 patients with type 2 diabetes mellitus (T2DM) were enrolled, of which 7 (26.9%) cases had adequate metabolic control (HbA1c < 7%) and 19 (73.1%) inadequate metabolic control (HbA1c ≥ 7%). It was observed that among patients with controlled T2DM, 2 (28.6%) cases presented good intake of fiber and 5 (71.4%) cases a regular intake. In contrast, in patients with uncontrolled T2DM, 13 (68.4%) patients reported a regular intake and 6 (31.6%) a poor intake. In relation to the identification of the gut microbiota, both groups presented a similar characterization. There were differences in the population of bacteria identified in both groups, however, the results were not statistically significant. The most frequently identified bacteria in controlled and uncontrolled T2DM patients were *Prevotella* (71.4% vs 52.6%), followed by *Firmicutes* (71.4% vs 42.1%), *Proteobacteria* (71.4% vs 36.8%) and *Bacteroidetes* (57.1% vs 37.8%). On the other hand, *Fusobacterium, Actinobacteria* were not identified in either of the two groups of study.

**Supplementary Information:**

The online version contains supplementary material available at 10.1186/s13104-021-05655-z.

## Introduction

Type 2 diabetes mellitus (T2DM) is a chronic metabolic disease characterized by progressive insulin resistance followed by deficit in insulin secretion. This condition causes the sustained elevation of plasma glucose, with subsequent increase in biochemical parameters such as glycosylated hemoglobin (HbA1c) [1, 2]. The prevalence of T2DM has increased in recent decades, affecting around 422 million people worldwide and accounting for the sixth leading cause of death in adults [3, 4]. Mortality is largely attributed to uncontrolled and decompensated diabetes, defined as levels of HbA1c greater than or equal to 7%, that generates different complications with difficult treatment [1–5].

The prevalence of T2DM in Latin America varies between 10 and 15%, which accounts for more than 23 million people [6]. In Peru it affects more than 2 million people, with an increasing trend over the years [7]. Different risk factors predispose individuals to suffer from this disease such as genetic susceptibility, anthropometric factors, lifestyle habits, endocrine system disorders, among others [8]. Recently, more attention has been focused on the relationship between metabolic diseases and microorganisms residing in the gastrointestinal tract, denominated as the gut microbiota. There is increasing evidence that the gut microbiota may have an important role regulating various biological functions, such as modulation of the innate and adaptative immune, metabolism, body homeostasis and nutrition [9–13].

Variations in the gut microbiota may arise from different factors related to diet, geographical area, medication, age, gender, among others [14, 15]. The quality and quantity of food intake by the host, particularly non-digestible carbohydrates and fiber, may modify the population of bacteria residing in the gut. Moreover, colonic bacteria ferment these substrates and produce short-chain fatty acids (SCFA), which are active metabolites that regulate metabolic and immune functions [16–18]. Alterations in the composition and function of the microbiota, denominated dysbiosis, can affect intestinal permeability and alter mucosa integrity. This process can produce an increase of monosaccharides absorption, synthesis of fatty acids on the liver and alter hormonal production in entero-endocrine cells [9, 10, 12, 19]. Recent studies have shown variations in the population of bacteria residing in the gut among healthy individuals and patients with T2DM, which have implications on pathogenesis and treatment of the disease [20, 21]. Bacteria belonging to the *Bacteroidetes, Firmicutes* and *Actinobacteria* genus predominate in healthy adults [22]. On the contrary, in people with metabolic disease there is greater presence of *Firmicutes* [23].

The objective of this study was to characterize the composition of the gut microbiota in type 2 Diabetes Mellitus (T2DM) patients with adequate and inadequate metabolic control, and its relationship with fiber consumption.

## Main text

### Study participants and sample collection

An observational, descriptive, cross-sectional study was carried out. The study design and the research procedure is illustrated in Additional file 1: Figure S1. Patients with a diagnosis of T2DM attending to the outpatient endocrinology service of the national hospital *Edgardo Rebagliati Martins* between August 2016 and February 2017 were enrolled. Patients were included if they signed informed consent to participate in the study and had a diagnosis of T2DM. Exclusion criteria were: a diagnosis of anemia, use of antibiotics in the previous 4 weeks and if they had changes in stool frequency and/or consistency (constipation or diarrhea) in the last 4 weeks. The patients were classified according to their control of the disease into: patients with adequate metabolic control those with HbA1c values < 7% and patients with inadequate metabolic control those participants with HbA1c values ≥ 7% [1, 2]. All patients who agreed to participate in the study were provided a sterile sample container to collect a fecal sample, which was subsequently stored at 4 °C and transported to the laboratory and stored at − 80 °C until analyzed.

### Clinical data collection

The clinical record was used to collect data regarding: weight, height, age, medical diagnosis, comorbidities associated with diabetes, duration of illness, medications and levels of glycosylated hemoglobin (HbA1c) taken in the previous 3 months. Moreover, a questionnaire was used to collect information such as: last gastrointestinal disease, frequency of defecation and data regarding food consumption. The analysis of the frequency of food consumption was based on the Block Dietary Screening Questionnaire for fat, fruit, vegetables and fiber intake of the Institute of Nutrition of Central America and Panama (INCAP). Patients were classified according to their fiber consumption into: good, regular and poor fiber intake.

### DNA amplification

A volume of 200 µl of fecal samples was processed for the extraction of bacterial DNA using the High Pure Template Preparation (Roche Applied Science, Penzberg Germany), according to the manufacturer’s instruction.

Polymerase Chain Reaction (PCR) was performed to amplify the genetic material using specific primers previously described by Murri et al [19] (Additional file 2: Table S1). The presence of 13 representative gut bacteria genus was analyzed: *Bacteroides, Fusobacterium, Actinobacteria, Eubacterium, Proteobacteria, Veillonella, Lactobacillus, Bifidobacterium, Clostridium, Firmicutes, Bacteroidetes, Enterocuccus* and *Prevotella*. Conditions of the PCR were an initial incubation of 95 °C for 5 min, followed by 45 cycles at 95 °C for 1 min; 52 °C for 45 s and 72 °C for 1 min; with a final extension at 72 °C for 10 min. The amplified products were later analyzed by gel electrophoresis on 2% agarose (FMC, Rockland, ME). Bacteria previously isolated and confirmed by automated sequencing were used as controls. All controls included in this study are disposable for scientific non-commercial purposes.

### Statistical analysis

A descriptive analysis was performed using the IBM Statistical Package for the Social Sciences (SPSS) software version 21.0 (Chicago, IL, USA). Frequencies and percentages of the study variables were calculated. To evaluate differences, the Fisher's exact test was used for proportions and the Student's T test for continuous variables. A p-value below 0.05 was considered statistically significant. Figures were created using the GraphPad Prism 9.1.0 program (San Diego, CA, USA).

### Ethics statement

This study was approved by the Research Ethics Board of the *Universidad Peruana de Ciencias Aplicadas,* Lima, Peru. The collection of the samples was done with a prior informed consent was obtained in written format from each participant. All samples were analyzed after a written informed consent was signed.

## Results

A total of 26 patients with type 2 diabetes mellitus were enrolled, with ages between 52 to 88 years. Patients were grouped according to the level of glycosylated hemoglobin (HbA1c): 7 (26.9%) cases had adequate metabolic control (HbA1c < 7%) and 19 (73.1%) had inadequate metabolic control (HbA1c ≥ 7%). The average body mass index (BMI) in uncontrolled diabetic patients was 28.8 kg/m2 considered by the World Health Organization as overweight, in addition these patients were the ones who presented a greater number of comorbidities. Similarly, in controlled patients an average BMI of 27.8 kg/m2 was found. Moreover, it was observed that the mean HbA1c was higher in the uncontrolled group (9.5%) compared to the controlled group (6.3%), being statistically significant (p < 0.01). Finally, patients with adequate control presented a higher frequency of hypertension (p = 0.04). The demographic and clinical characteristics of the population are described in Table 1.

**Table 1:** Demographic and clinical data of controlled and uncontrolled T2DM

Demographic characteristics	Total participants (n = 26)	Controlled T2DM (n = 7)	Uncontrolled T2DM (n = 19)	P value
Male (%)	16 (62.0%)	4 (57.0%)	12 (63.0%)	0.8
Female (%)	10 (37.0%)	3 (43.0%)	7 (37.0%)	0.9
Mean age (SD)*	66.1 (± 9.9)	68.3 (± 9.7)	65.3 (± 10.2)	0.5
Clinical data				
Overweight/obesity (%)	15 (57.7%)	3 (42.8%)	12 (63.0%)	0.5
Mean HbA1c (SD)**	8.9 (± 2.4)	6.3 (± 0.3)	9.5 (± 2.3)	< 0.01
Mean BMI (SD)***	28.5 (± 4.8)	27.9 (± 5.0)	28.8 (± 4.8)	0.7
Mean time since disease onset (SD)*	18.7 (± 10.7)	16.5 (± 9.2)	19.5 (± 11.3)	0.5
Dyslypidemia (%)	10 (38.5%)	2 (28.3%)	8 (42.0%)	0.7
Hypertension (%)	18 (69.2%)	7 (100%)	11 (57.0%)	0.04
Retinopathy (%)	6 (23.0%)	0 (0.0%)	6 (31.6%)	–
Neuropathy (%)	13 (50.0%)	2 (28.6%)	11 (57.9%)	0.4

*Mean in years, **in percentage (%), ***in Kg/m^2^

An evaluation of the medications used was performed. In the group of patients with metabolic control, 4 (57.1%) were taking metformin; 1 (14.3%), metformin and insulin and 2 (28.6%) cases reported no medication for glycemic control. In the group of patients with inadequate metabolic control, 12 (63.1%) were taking only metformin; 5 (26.3%), metformin and insulin, and 2 (10.5%) only insulin. Table 2 shows the evaluation of the dietary fiber intake in the two groups; however, no statistical differences were found. It was observed that among patients with controlled T2DM, 2 (28.6%) cases presented good intake of fiber and 5 (71.4%) cases a regular intake. In contrast, in patients with uncontrolled T2DM, 13 (68.4%) patients reported a regular intake and 6 (31.6%) a poor intake. Similarly, it was found that 85.8% of patients with adequate metabolic control consume yogurt with pre and probiotics at least once a week, while 73.7% of uncontrolled patients consume this product with the same frequency.

**Table 2:** Frequency of dietary fiber consumption

Food type	Controlled T2DM (n = 7)				Uncontrolled T2DM (n = 19)			
	Frequency				Frequency			
	Daily	Weekly	Monthly	Never	Daily	Weekly	Monthly	Never
Cereals	1	3	2	1	2	5	6	6
Legumes	0	6	0	1	1	15	3	0
Vegetables	6	1	0	0	5	14	0	0
Fruits	4	3	0	0	5	11	3	0
Whole.grain products	3	1	0	3	11	2	1	5
Oilseeds	1	5	0	1	2	9	4	4

**Fig. 1:**
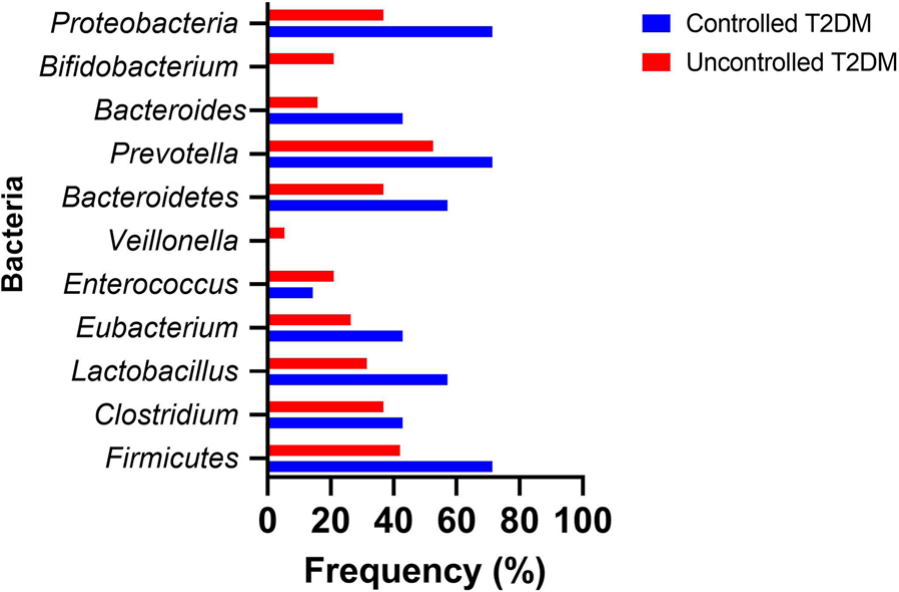
Microbiota in T2DM patients

In relation to the identification of the gut microbiota, both groups presented a similar characterization as shown in Fig. 1. There were differences in the population of bacteria identified in both groups, however the results were not statistically significant. The most frequently identified bacteria in controlled and uncontrolled T2DM patients were *Prevotella* (71.4% vs 52.6%), followed by *Firmicutes* (71.4% vs 42.1%), *Proteobacteria* (71.4% vs 36.8%) and *Bacteroidetes* (57.1% vs 37.8%). On the other hand, *Fusobacterium, Actinobacteria* were not identified in either of the two study groups (Additional file 2: Table S2).

## Discussion

In the present study, we compared the gut microbiota of patients with a diagnosis of type 2 Diabetes Mellitus (T2DM) with adequate metabolic control and patients with inadequate metabolic control. Previous studies highlight the close relationship between the intestinal microbiota and T2DM, with a predominant role of these microorganisms in the pathogenesis of this disease [15, 23].

In our study, *Prevotella, Firmicutes, Proteobacteria* and *Bacteroidetes* were the predominant genus observed in both groups of patients, with greater frequency of detection in the metabolic controlled group. Currently, few studies have focused on differences in the gut microbiota among controlled and uncontrolled patients with T2DM, with most studies comparing the bacteria population between T2DM and healthy subjects. Previous studies found that the population of *Firmicutes* and *Clostridia* have a significant reduction in patients with T2DM compared to healthy subjects [20]. It could be expected that patients with adequate metabolic control possess a similar bacteria profile compared to healthy subjects, however, we found similar bacterial profiles between our study groups. Moreover, it has been reported that, *Firmicutes* and *Eubacterium* are two pivotal bacteria present in the gut microbiota, which have the ability to metabolize indigestible polysaccharides and increase the production of short chain fatty acids (SCFA). These molecules are absorbed by the body and produce an increase in calorie gain [8–10, 24–27]. Therefore, we can infer that if there is a greater proportion of these bacterial genus, overweight and obesity are more likely to arise [26, 27]. In this study it was observed that 7 (53.8%) patients with the presence of *Firmicutes* and *Eubacterium* were overweight or obese; among these, 1 case had adequate metabolic control and 6 cases had inadequate metabolic control. On the other hand, it has been previously reported that the *Bacteroides/Prevotella* ratio is elevated in patients with T2DM, which contrasts with our results. These findings may be explained by several factors that can modify the gut microbiota such as: dietary habits, genetic predisposition and different regions of study [27].

The intake of non-digestible carbohydrates and fiber has been tightly related to the modulation of the gut microbiota. For example, a study showed that 49 obese subjects were exposed to an increase in dietary fiber, which produced an increase in the presence of *Bifidobacterium* and *Lactobacillus*, on the other hand, *Clostridium* and *Enterococcus* were reduced [27, 28]. There is also evidence that the degradation of fiber by gut bacteria releases butyrate, a SCFA which alters the absorption and permeability of the intestinal mucosa [18, 20]. Moreover, this molecule can also help reduce pro-inflammatory cytokines, enhance insuline tolerance and decrease glygosylated hemoglobin levels [28]. Finally, butyrate can contribute to modulate serotonine levels, which is involved in satiety regulation [24, 26]. In our study, we could observe that a higher number of patients with controlled disease presented an adequate and regular intake of fiber. In contrast, more patients with uncontrolled disease had a poor intake of fiber.

Other factors may also have a role in the regulation of the gut microbiota, for example hypocaloric diet with probiotics, prebiotics and polyphenols has been associated with an increase in *Bacteroidetes, Prevotella* and *Enterococcus*, which reduce expression of lipopolysaccharides and inflammation [29]. It has been reported that the use of metformin may modulate the gut microbiota, increasing the population of *Lactobacillus* and *Bifidobacterium* [14, 30]. In our study, we could identify the presence of *Lactobacillus* in three of the four patients with adequate metabolic control receiving metformin and no cases of *Bifidobacterium*. Meanwhile, in the group with uncontrolled diabetes, we could find Lactobacillus in 5 (29.4%) cases and *Bifidobacterium* in 6 (35.3%).

In conclusion, *Firmicutes, Prevotella, Proteobacteria* and *Bacteroidetes* were the most frequently bacteria identified in both groups, with a slightly greater detection in the adequate metabolic control group. *Actinobacteria* and *Fusobacterium* were not identified in either groups. The intake of fiber was higher in patients with controlled T2DM, which may be related to the maintenance of the gut microbiota and control of the disease.

## Limitations

Firstly, the small sample size used in the current study was the main limitation, given that some of our findings did not attain statistical significance. Another important limitation is that we could only determine the presence or absence, and not the relative abundance of each specific bacteria. Also recall bias may be a limitation, given that patients were asked to provide self-reported information on dietary habits. Because of the study design it could not be determined if changes in the microbiota correspond to the metabolic disease or the other way around. Further longitudinal studies are required to provide a better characterization between metabolic control, microbiota and diet given the increasing prevalence of type 2 Diabetes Mellitus.

## Supplementary Information

**Additional file1:** **Figure S1.** Flowchart of the research methods and study design.

**Additional file2: Table S1.** Primers and sequences used for the molecular identification of the gut microbiota. Adapted from Murri et al [19]. **Table S2.** Genus of bacteria found by group of diabetic patients.

## Data Availability

Abstraction format used in the study and dataset are available and accessible from the corresponding author upon request in the link: https://figshare.com/s/811314b6ad52e8abb8fc

## Abbreviations

T2DM: Type 2 diabetes mellitus
HbA1c: Glycosylated hemoglobin
SCFA: Short-chain fatty acids
PCR: Polymerase chain reaction
DNA: Deoxyribonucleic acid

## References

[1] Sonne DP, Hemmingsen B, American Diabetes Association (2017). Standards of medical care in diabetes: 2017. Diabetes Care.

[2] Roden M (2016). Diabetes mellitus: definition, classification and diagnosis. Wien Klin Wochenschr.

[3] Organización Mundial de la Salud (OMS) | Diabetes. OMS. [citado 4 de junio de 2017]. Recuperado a partir de: http://www.who.int/mediacentre/factsheets/fs312/es/.

[4] Organización Mundial de la Salud (OMS). Informe mundial sobre la diabetes. Ginebra, 2016.

[5] Chen L, Magliano DJ, Zimmet PZ (2012). The worldwide epidemiology of type 2 diabetes mellitus—present and future perspectives. Nat Rev Endocrinol.

[6] Asociación Latinoamericana de Diabetes (ALAD). Guías ALAD de diagnóstico, control y tratamiento de la Diabetes Mellitus tipo 2. Revista A; 2013. http://www.bvs.hn/Honduras/UICFCM/Diabetes/GUIAS_ALAD_2013.pdf. Accessed 11 Nov 2020.

[7] Organización Mundial de la Salud (OMS). Perfiles de los países para la diabetes. 2016. Recuperado a partir de: http://www.who.int/diabetes/countryprofiles/per_es.pdf?ua=1. Accessed 15 July 2020.

[8] Everard A, Cani PD (2013). Diabetes, obesity and gut microbiota. Best Pract Res Clin Gastroenterol.

[9] Robles-Alonso V, Guarner F (2013). Progreso en el conocimiento de la microbiota intestinal humana. Nutr Hosp.

[10] Icaza-Chávez M (2013). Microbiota en la salud y la enfermedad. Rev Gastroenterol Mex.

[11] Musso G, Gambino R, Cassader M (2011). Interactions between gut microbiota and host metabolism predisposing to obesity and diabetes. Annu Rev Med.

[12] Giglio ND, Burgos F, Cavagnari BM (2013). Microbiota intestinal: sus repercusiones clínicas en el cuerpo humano. Arch Argent Pediatría.

[13] Tinahones F. La importancia de la microbiota en la obesidad. Rev Esp Endocrinol Pediatr. 2017, S8

[14] Brunkwall L, Orho-Melander M (2017). The gut microbiome as a target for prevention and treatment of hyperglycaemia in type 2 diabetes: from current human evidence to future possibilities. Diabetologia.

[15] Pushpanathan P, Mathew GS, Selvarajan S, Seshadri KG, Srikanth P (2019). Gut microbiota and its mysteries. Indian J Med Microbiol.

[16] Walker AW, Ince J, Duncan SH, Webster LM, Holtrop G, Ze X, Brown D, Stares MD, Scott P, Bergerat A, Louis P, McIntosh F, Johnstone AM, Lobley GE, Parkhill J, Flint HJ (2011). Dominant and diet-responsive groups of bacteria within the human colonic microbiota. ISME J.

[17] Flint HJ (2012). The impact of nutrition on the human microbiome. Nutr Rev.

[18] Richards J, Yap Y, McLeod K, Mackay C, Mariño E (2016). Dietary metabolites and the gut microbiota: an alternative approach to control inflammatory and autoimmune diseases. Clin Transl Immunol.

[19] Devaraj S, Hemarajata P, Versalovic J (2013). La microbiota intestinal humana y el metabolismo corporal: implicaciones con la obesidad y la diabetes. Acta Bioquím Clín Latinoam.

[20] Larsen N, Vogensen FK, van den Berg FWJ, Nielsen DS, Andreasen AS, Pedersen BK (2010). Gut microbiota in human adults with type 2 diabetes differs from non-diabetic adults. PLoS ONE.

[21] Murri M, Leiva I, Gomez-Zumaquero JM, Tinahones FJ, Cardona F, Soriguer F, Queipo-Ortuño MI (2013). Gut microbiota in children with type 1 diabetes differs from that in healthy children: a case-control study. BMC Med.

[22] Ferreira A, Boroni AP, Diniz D, Gouveia MC, Salles T. Microbiota gastrintestinal. Evidências da sua influência na saúde e na doença. 1ra ed. Rio de Janeiro: Rubio; 2015, 264p.

[23] Rinninella E, Raoul P, Cintoni M, Franceschi F, Miggiano GAD, Gasbarrini A, Mele MC (2019). What is the healthy gut microbiota composition? A changing ecosystem across age, environment, diet, and diseases. Microorganisms.

[24] Munoz-Garach A (2016). Microbiota y diabetes mellitus tipo 2. Endocrinol Nutr.

[25] Tilg H, Moschen AR (2014). Microbiota and diabetes: an evolving relationship. Gut.

[26] El GM (2013). papel de la microbiota intestinal en el desarrollo de la obesidad y de la diabetes de tipo-2. Rev Chil Endocrinol Diabetes.

[27] Holmes E, Li JV, Marchesi JR, Nicholson JK (2012). Gut microbiota composition and activity in relation to host metabolic phenotype and disease risk. Cell Metab.

[28] Singh RK, Chang HW, Yan D (2017). Influence of diet on the gut microbiome and implications for human health. J Transl Med.

[29] Etxeberria U, Milagro FI, González-Navarro CJ, Martínez JA (2016). Papel en la obesidad de la microbiota intestinal.

[30] Wu H, Esteve E, Tremaroli V, Khan MT, Caesar R, Mannerås-Holm L, Ståhlman M, Olsson LM, Serino M, Planas-Fèlix M, Xifra G, Mercader JM, Torrents D, Burcelin R, Ricart W, Perkins R, Fernàndez-Real JM, Bäckhed F (2017). Metformin alters the gut microbiome of individuals with treatment-naive type 2 diabetes, contributing to the therapeutic effects of the drug. Nat Med.

