# Supplementary figures and images for: Characterization of the gut microbiota in diabetes mellitus II patients with adequate and inadequate metabolic control
Source: BMC Res Notes. 2021 Jun 24;14:238. doi: 10.1186/s13104-021-05655-z (PMC8223318; doi:10.1186/s13104-021-05655-z)

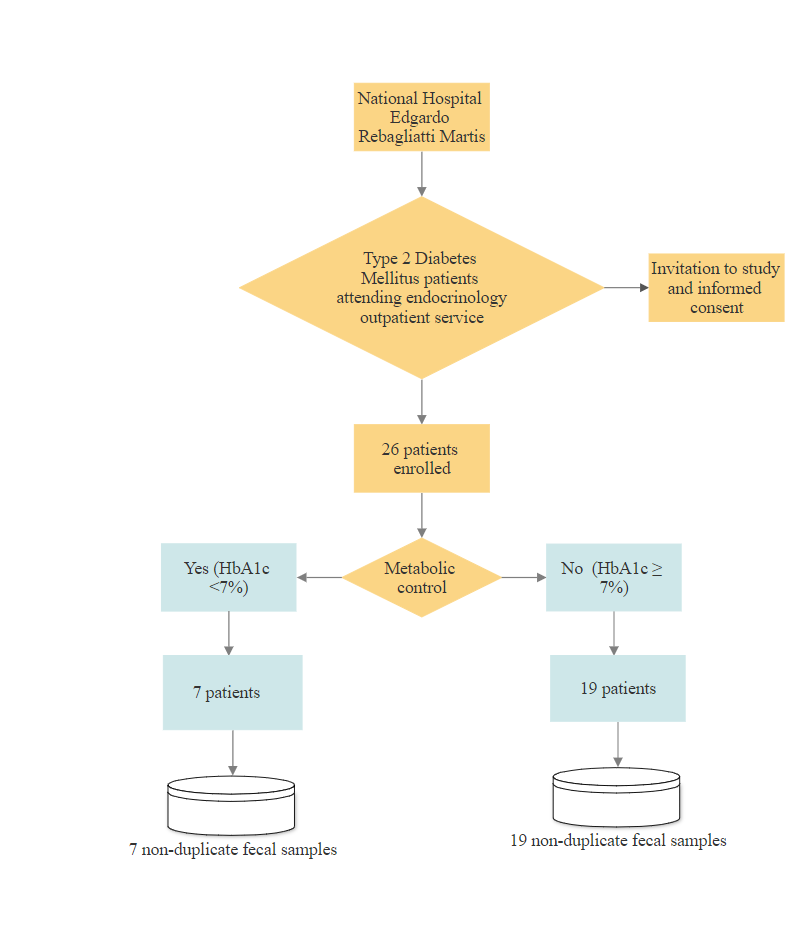

Supplement: Supplementary file 1 — Additional file1: Figure S1. Flowchart of the research methods and study design. [file 13104_2021_5655_MOESM1_ESM.png]
