# Supplementary material for: Characterization of the gut microbiota in diabetes mellitus II patients with adequate and inadequate metabolic control
Source: BMC Res Notes. 2021 Jun 24;14:238. doi: 10.1186/s13104-021-05655-z (PMC8223318; doi:10.1186/s13104-021-05655-z)
Supplement: Supplementary file 2 — Additional file2: Table S1. Primers and sequences used for the molecular identification of the gut microbiota. Adapted from Murri et al [19]. Table S2. Genus of bacteria found by group of diabetic patients. [file 13104_2021_5655_MOESM2_ESM.docx]

**Table S1.** Primers and sequences used for the molecular identification of the gut microbiota. Adapted from Murri et al. [19]

| **Assay** | **Bacteria** | **Primers** | **Oligonucleotide sequence 5’ - 3’** | **Amplicon size (base pair)** | **Annealing temperature (ºC)** | **Melting temperature (°C)** | **Access number** |
| --- | --- | --- | --- | --- | --- | --- | --- |
| Multiplex n°1 | *Bacteroides*  (Layton 2006) | BactF (Bact1) | GAGAGGAAGGTCCCCCAC | 106 | 52 | 60.7 | MN435123 |
|  |  | BactR (Bact2) | CGCTACTTGGCTGGTTCAG |  |  | 59.5 |  |
|  | *Proteobacteria*  (Bartosch 2004) | ProteoF (Prot1) | CATGACGTTACCCGCAGAAGAAG | 195 |  | 64.7 | JN838219 |
|  |  | ProteoR (Prot2) | CTCTACGAGACTCAAGCTTGC |  |  | 61.3 |  |
|  | *Fusobacterium* | FusoF | CCCTTCAGTGCCGCAGT | 273 |  | 57.2 | MF185959 |
|  |  | FusoR | GTCGCAGGATGTCAAGAC |  |  | 56.1 |  |
|  | *Eubacterium* | BlautF | CGGTACCTGACTAAGAAGC | 429 |  | 57.3 | MW341148 |
|  |  | BlautR | AGTTTCATTCTTGCGAACG |  |  | 53.0 |  |
|  | *Actinobacteria* | ActinoF | CGCGGCCTATCAGCTTGTTG | 600 |  | 62.5 | MW931624 |
|  |  | ActinoR | CCGTACTCCCCAGGCGGGG |  |  | 68.1 |  |
| Multiplex n°2 | *Lactobacillus*  (Delroisse 2008) | LactoF | GAGGCAGCAGTAGGGAATCTTC | 126 | 52 | 64.0 | MW672536 |
|  |  | LactoR | GGCCAGTTACTACCTCTATCCTTCTTC |  |  | 68.3 |  |
|  | *Clostridium* | ClociF | GCACAAGCAGTGGAGT | 239 |  | 50.7 | LT960605 |
|  |  | ClociR | CTTCCTCCGTTTTGTCAA |  |  | 51.6 |  |
|  | *Veillonella* | VeilF | ACCAACCTGCCCTTCAGA | 343 |  | 56.1 | HF675187 |
|  |  | VeilR | CGTCCCGATTAACAGAGCTT |  |  | 58.4 |  |
|  | *Bifidobacterium* | BifidF | CTCCTGGAAACGGGTGG | 550 |  | 57.2 | MW678774 |
|  |  | BifidR | GGTGTT CTTCCCG ATATCTACA |  |  | 60.3 |  |
| Individual assay | *Prevotella* | PrevF | GGTTCTGAGAGGAAGGTCCCC | 121 | 52 | 65.3 | MW570878 |
|  |  | PrevR | TCCTGCACGCTACTTGGCTG |  |  | 62.5 |  |
| Individual assay | *Bacteroidetes*  (Guo 2008) | BacTTF | CATGTGGTTTAATTCGATGAT | 126 | 52 | 53.5 | MW876299 |
|  |  | BacTTR | AGCTGACGACAACCATGCAG |  |  | 60.5 |  |
| Individual assay | *Firmicutes*  (Guo 2008) | FirmiF | ATGTGGTTTAATTCGAAGCA | 126 | 52 | 52.3 | MZ067738 |
|  |  | FirmiR | AGCTGACGACAACCATGCAC |  |  | 60.5 |  |
| Individual assay | *Enterococcus* | EnteF (Ente1) | CCCTTATTGTTAGTTGCCATCATT | 144 | 52 | 60.1 |  |
|  |  | EnteR (Ente2) | ACTCGTTCTTCCCATGT |  |  | 50.0 |  |

**Table S2.** Bacteria genus identified in patients with T2DM.

| Bacteria genus | Total patients  (n=26) | Patients with controlled T2DM (n=7) | Patients with uncontrolled T2DM (n=19) |
| --- | --- | --- | --- |
| *Firmicutes* | 13 (50.0%) | 5 (71.4%) | 8 (42.1%) |
| *Clostridium* | 10 (38.5%) | 3 (42.9%) | 7 (36.8%) |
| *Lactobacillus* | 10 (38.5%) | 4 (57.1%) | 6 (31.6%) |
| *Eubacterium* | 8 (30.8%) | 3 (42.9%) | 5 (26.3%) |
| *Enterococcus* | 5 (19.2%) | 1 (14.3%) | 4 (21.0%) |
| *Veillonella* | 1 (3.8%) | 0 (0.0%) | 1 (5.3%) |
| *Bacteroidetes* | 11 (42.3%) | 4 (57.1%) | 7 (36.8%) |
| *Prevotella* | 15 (57.7%) | 5 (71.4%) | 10 (52.6%) |
| *Bacteroides* | 6 (23.1%) | 3 (42.9%) | 3 (15.8%) |
| *Actinobacteria* | 0 (0.0%) | 0 (0.0%) | 0 (0.0%) |
| *Bifidobacterium* | 4 (15.4%) | 0 (0.0%) | 4 (21.0%) |
| *Proteobacteria* | 12 (46.1%) | 5 (71.4%) | 7 (36.8%) |
| *Fusobacterium* | 0 (0.0%) | 0 (0.0%) | 0 (0.0%) |
